# Empowering Communities: Tailored Pandemic Data Visualization for Varied Tasks and Users

**DOI:** 10.1109/MCG.2024.3509293

**Published:** 2025

**Authors:** Tom Baumgartl, Mohammad Ghoniem, Tatiana von Landesberger, G. Elisabeta Marai, Silvia Miksch, Sibylle Mohr, Simone Scheithauer, Nikita Srivastava

**Affiliations:** University of Cologne, 50923, Cologne, Germany; Luxembourg Institute of Science and Technology, L-4362, Esch-sur-Alzette, Luxembourg; University of Cologne, 50923, Cologne, Germany; University of Illinois Chicago, Chicago, IL, 60607, USA; TU Wien, A-1040, Vienna, Austria; University of Glasgow, G12 8QQ, Glasgow, U.K.; University Medical Center, 37075, Göttingen, Germany; University Medical Center, 37075, Göttingen, Germany

## Abstract

Data visualization methodologies were intensively leveraged during the COVID-19 pandemic. We review our design experience working on a set of interdisciplinary COVID-19 pandemic projects. We describe the challenges we met in these projects, characterize the respective user communities, the goals and tasks we supported, and the data types and visual media we worked with. Furthermore, we instantiate these characterizations in a series of case studies. Finally, we describe the visual analysis lessons we learned, considering future pandemics.

The COVID-19 pandemic generated a torrent of data from numerous and varied sources: from case incidence reports from medical centers to mathematical models from the scientific community. Diverse communities, including biomedical researchers, decision makers, journalists, and the public were looking for insight into these data (Figure 1). Data visualization was rapidly leveraged as an investigative tool.

At the same time, challenges hindered the process of creating informative data visualizations: there was time pressure, limiting the available time for design exploration and client feedback; the data kept streaming, without prior knowledge of its ultimate size or range of values; there were literacy and communication barriers, and limits to personnel, infrastructure, and applicable teamwork methodologies.

We are eight researchers in the biomedical and computing field (6 women, 2 men) who worked in medical centers and in academia on COVID-19 related projects that leveraged data visualization. We all met to reflect on our experience at the Dagstuhl-24019 seminar Reflections on Pandemic Visualization in February 2024.

Our backgrounds include infectious disease modeling (2), data science (1), and data visualization research (5), with 1 to 25+ years of professional experience. We worked on a range of COVID-19 related projects, nine located in the European Union (four in Germany, four in Luxembourg, one in Austria), one in Scotland, U.K., and one in the USA. These projects started in early 2020, with some building on earlier work, and most had completed by 2024, although some are still ongoing.

In this work, we briefly describe a few of these projects, then systematically investigate and document the data visualization needs of the different communities, the goals and tasks we needed to support, and the data types and visual media we worked with during the pandemic. We briefly sum up the lessons we learned throughout this experience, so that future data visualization practitioners can hit the ground running in the next emergency.

## PROJECTS

In this section, we briefly describe a small subset of the pandemic projects we have worked on, to provide a sense of the variety of clients, data types, goals, and desired formats we met.

### COVis: Analyzing Event Data in Journalism

**FIGURE 2.**
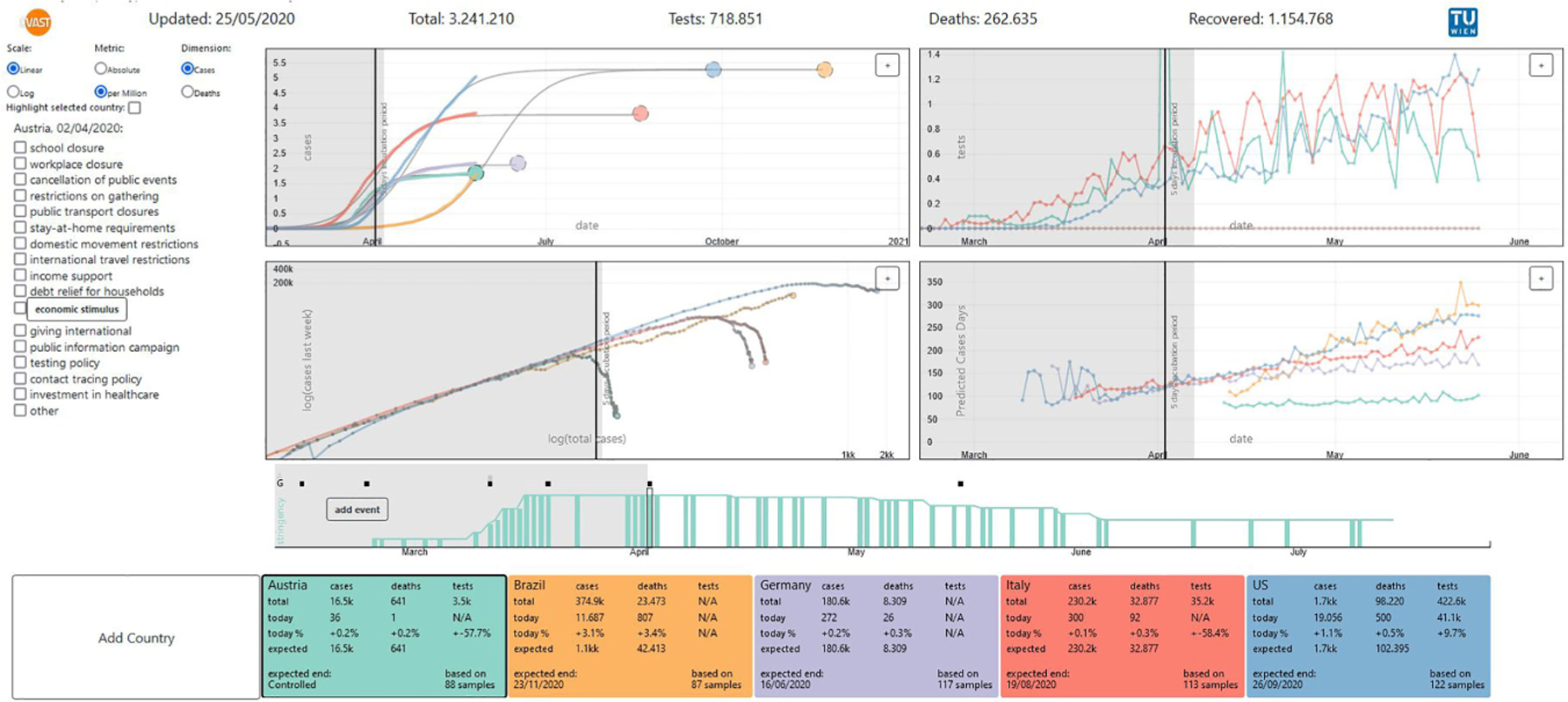
COVis’s charts display the temporal evolution of actual/projected cases/deaths, tests, and total cases/deaths × last week’s cases/deaths. The event panel displays information and source references. The events time chart presents policy changes over time. Countries can be excluded or included into analysis.

The TU Wien group teamed up with a journalist from Brazil to analyze publicly available COVID-19 data. COVis^9^ (Figure 2) combines temporal aspects of COVID-19 data with a predictive model to estimate the spread of the disease in various scenarios, and to correlate and monitor the virus spread in relation to different government responses. COVis was a voluntary, remote, open source project. It aimed to inform journalists and the public, and resulted in an exploratory visualization and data-driven storytelling system. Our design rationale was to use simple and intuitive visual means that journalists and the public are familiar with.

As shown in Table 1, we (SM1*’*s group) targeted journalists and the public, and we used core statistics (e.g., confirmed cases, number of tests, and death toll for each country). A second dataset, from Oxford University, contained the dates and news sources for public measures, such as school closures, together with stringency levels, giving our approach an event-based dimension and links to source for investigation. These data were integrated with projections from a logistic regression model. Following the *“*5 Ws*”* principle (Who, What, Where, When, Why), we address the following tasks: 1) Identify different country behavior (Who? and Where?); 2) Identify event influence (What?); 3) Perceive variables change over time (When?); and 4) Reason about changes of data pattern (Why?).

### CONTACT: The Contact Tracing Model

The Scottish Covid Response Consortium (SCRC) adapted existing large-scale animal disease models to assess medium- and long-term strategies for controlling the COVID-19 pandemic. The project took the form of a visual analytics tool using representative trees and other basic encodings to inspect transmission patterns.

The Contact Tracing model, an individual-based stochastic epidemiological model, simulates disease spread over dynamic contact networks to find optimal contact tracing- and isolation policies while minimizing the burden of contact tracing. Visualizations support the epidemiological modeling process at various stages: from understanding and fitting disease-specific parameters to checking model fitness and outputs, to communicating policy-specific results to various stakeholders and audiences.^3,4^

**FIGURE 3.**
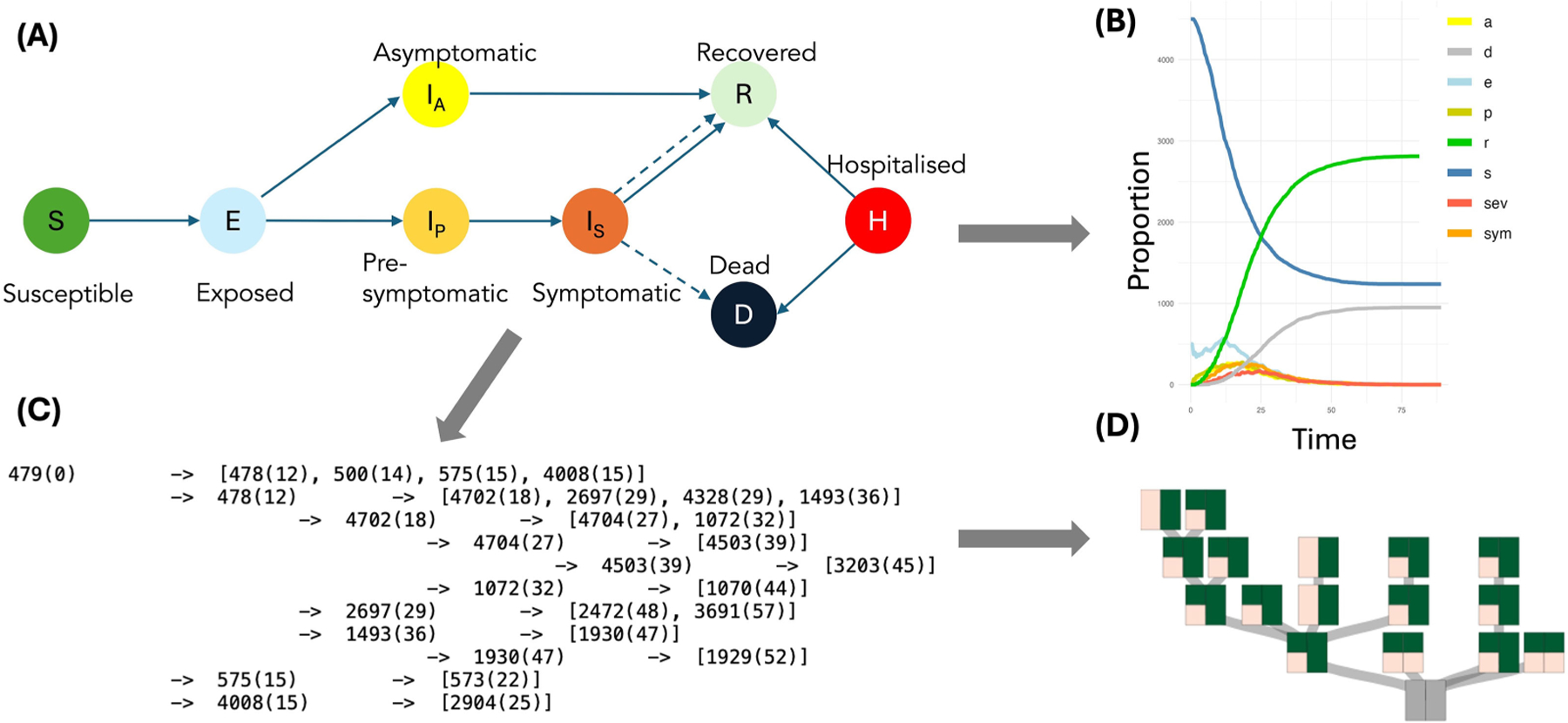
(A) Contact Tracing Model: Epidemiological compartments. (B) Example output at the population scale for a simulated policy. (C) Example of model output of complex transmission chains at the individual scale at time (t). (D) Comparison of impact of two control policies on individual transmission chains using representative trees.^3^ Trees are split into two sides to represent individual policies; green colors indicate the proportions of prevented infection routes.

The project (SM2*’*s group) targeted infectious disease modelers, public health experts, policy-makers, and advisory groups (see Table 1). It used simulation outputs generated by a compartmental model, spreading the infection on a dynamic contact network in different settings. Outputs include standard epidemiological summary statistics at the population level (number of susceptible, exposed, infected, removed individuals at time and complex individual transmission trees of potential infectious contacts at time and location (Figure 3). Tailored visualization of individual transmission patterns was used to understand the number of individuals under quarantine, the duration of their quarantine (so-called *“*test-to-release*”* policies), how many infections were prevented by adapting respective contact tracing policies, and to effectively communicate results to various audiences. This level of detail is rarely used or visualized in standard epidemiological research. We showed that it can help balancing the effectiveness of complex contact-tracing control policies to control spread through the population while keeping disruption to the individual at a minimum.

### MOTIV: Measuring the Effect of Pandemic Measures

**FIGURE 4.**
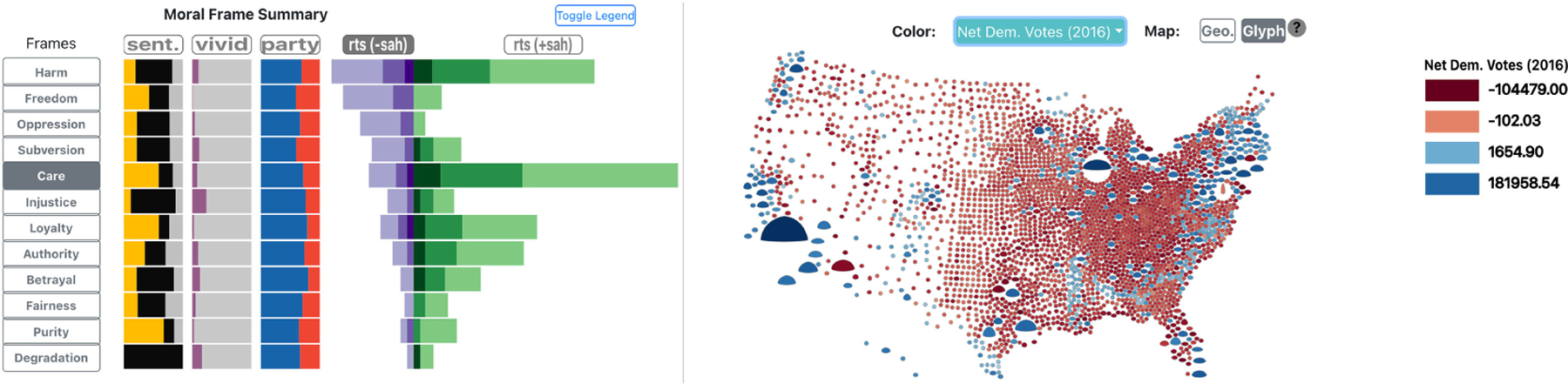
Custom encodings in MOTIV, a web application for analyzing the U.S. population response to Stay-at-Home (SAH) orders from a moral frame perspective, based on social media. (Left) Barcharts show the distribution of moral frames, with Care being most predominant. (Right) Custom glyphs show the distribution of electoral votes along with SAH support across the U.S.

This U.S.-funded project (NSF-IIS-2031095) analyzed the population response to stay-at-home orders using the *moral frame theory* and an inference model, both informed by social media, pandemic, demographic, socioeconomic, and political context data^1^ (Figure 4). We (GEM*’*s group) ran this open source project remotely from early 2020 to 2022. The result was an interactive web application, from which screenshots were later distributed in print and in presentations.

As shown in Table 1, the project targeted communication researchers with expertise in media analysis and race relations, with the further goal of providing city, state, and federal administrations with insight into the efficacy of specific measures (i.e., Stay-at-Home orders). To this end, the project analyzed health data over space and time: COVID-19 case numbers, at the county and city level; and health-related behaviors such as self-reported mask usage. It also looked at demographic and socioeconomic data from the U.S. Census, and the sentimental and moral content of tweets about stay-at-home orders. Last, it examined causal inferences among the different data variables.

The specific tasks were pandemic-stage centered and temporal analysis oriented, and included: understanding the demographic and political context of the population response to the measures; evaluating the effect of stay-at-home measures as reflected in tweets; leveraging a specific inference model interactively; and communicating the findings to other communications and causal inference researchers. Due to limited visual literacy in the client group, the project used basic and simple custom encodings.

Through much data foraging work, we generated valuable insights, and noticed a higher acceptance of high data density layouts among our collaborators.

### B-FAST: Infection Transmission in Hospitals

**FIGURE 5.**
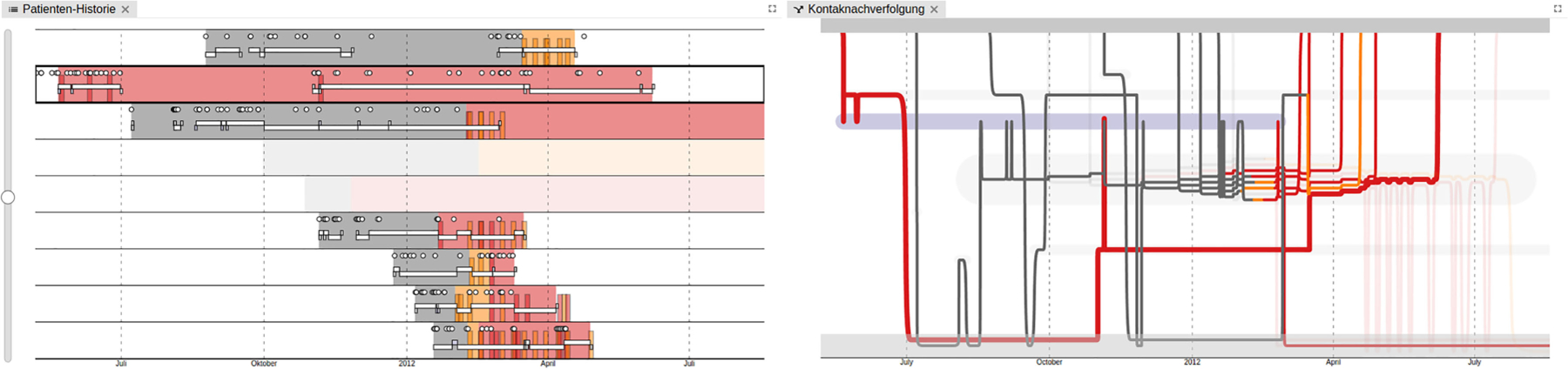
Part of the SmICS Visualization Dashboard. (Left) Timeline of individual patients, showing their infection status, test results, and ward stays. (Right) Storyline visualization of individual patients, encoding patient contacts as line bundles to show possible transmission pathways via patient contact.^2^

The Infection Control Use Case of the 2018 HiGHmed project developed a system for early outbreak detection of bacterial pathogens, funded by the German Federal Ministry of Education and Research (BMBF-01KX2021). In early 2020, the B-FAST project adapted this system for COVID-19^2^ (Figure 5). B-FAST aims to provide nationwide applied surveillance and testing information, it was deployed in participating hospitals, and it is integrated with the NUM (*“*Netzwerk Universitätsmedizin*”*) initiative, also funded by BMBF. The interactive web application of the Smart Infection Control System (SmICS) Dashboard is open source.

As shown in Table 1, we (group of TB and TvL) targeted infection prevention and control; hygiene experts/hospital epidemiologists, and other healthcare professionals. We visualized time-dependent patient data, including vaccinations, ward stays, and diagnoses. Time data are highly uncertain. Experts recommended not visually encoding these uncertainties, though users are aware of them.

The main objective of the standard SmICS from the Infection Control Use Case was to identify potential transmission events that lead to outbreaks at a very early time point to decrease the risk of an outbreak or the number of affected patients in the hospital.

Through close collaboration and an iterative design process with domain experts, the resulting system accelerated outbreak analysis in infection control.

### HEADS: Human Emission of Aerosol and Droplet Statistics

**FIGURE 6.**
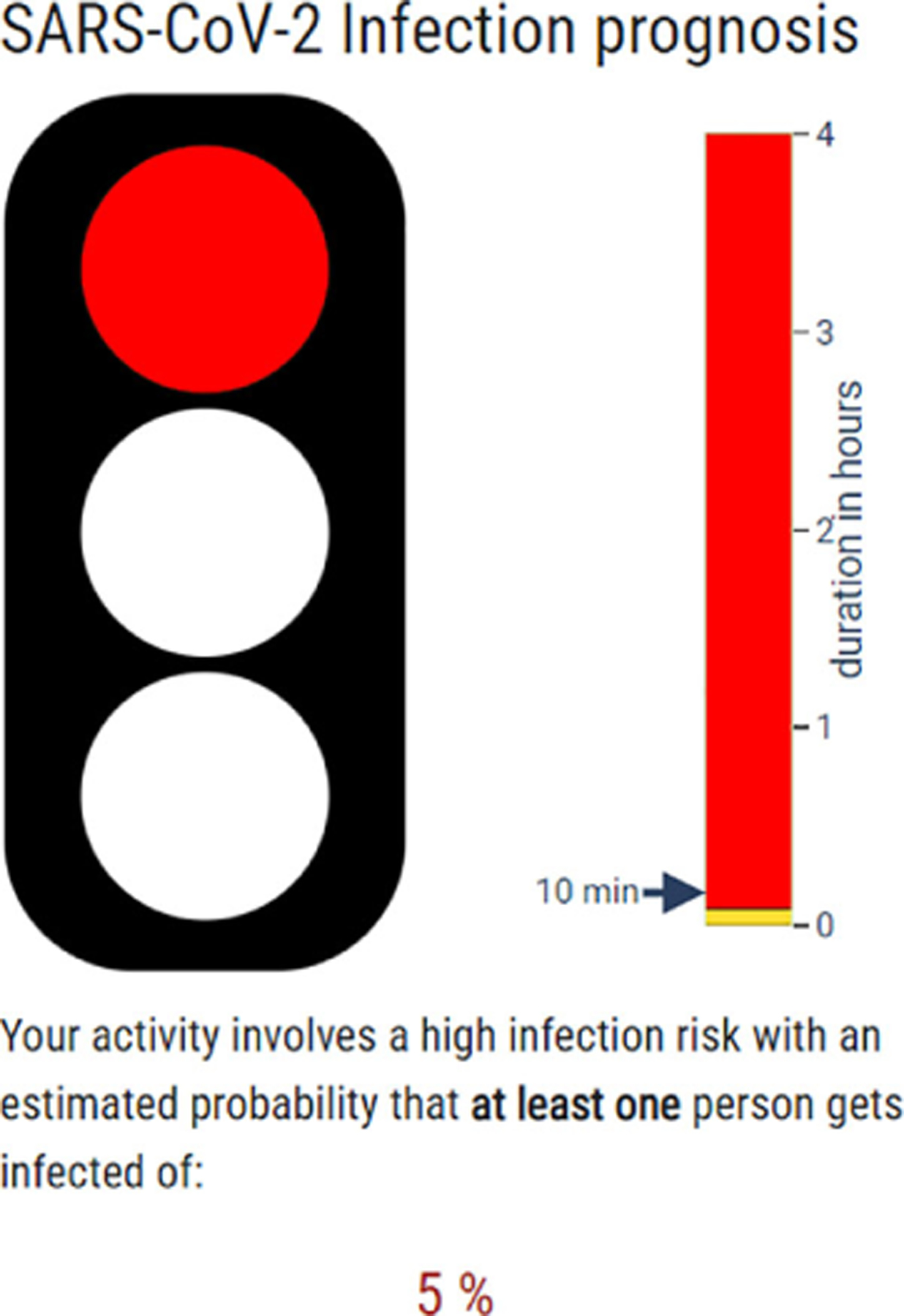
SARS-CoV-2 Infection Prognosis for High-Risk Activities: The red traffic light visual indicates a high infection risk related to activities lasting over 10 min, with a 5% chance of at least one person becoming infected.

The HEADS app, developed by the Max Planck Institute for Dynamics and Self-Organization with the Institute of Infection Control and Infectious Diseases, and funded by BMBF-01KX2021, offers data on aerosol and droplet emissions. The project aimed to help researchers, public health officials, policymakers, and the public to understand particle emission dynamics, vital for disease control. It took the shape of an interactive app with an intuitive traffic light metaphor (Figure 6), and it used data from experiments and literature to model the spread of infectious aerosols.^10^

As shown in Table 1, it used a large number of aerosol, virus, and environmental parameters, line chart encodings, and it aimed to assess the probability of airborne disease transmission in different settings, understand how various factors such as mask usage affect infection risk, model the spread of infectious aerosols over time and space, and inform public health interventions and personal safety measures.

## USER GROUPS

In this section and below, we systematically analyze the client types, data types, goals, and media in the projects described in this work, and augment this analysis with additional insights based on the totality of projects we worked on during the pandemic. Our goal is to help future collaborative efforts in developing effective visual solutions.

In the biomedical field, the COVID-19 stakeholders were diverse. (a) Physicians, clinical, and lab researchers from various biomedical fields were involved in direct patient care and crisis management. (b) Policymakers at city, state, and federal levels also played a crucial role in mitigating the virus spread, protecting public health, ensuring healthcare system readiness, and supporting the economy. This highlights the need for tailored visualizations to communicate complex data to policymakers, aiding informed decision-making and strategic planning. (c) Public-facing advisors such as data journalists, newscasters, and other media professionals were vital in disseminating accurate information, countering misinformation, and educating the public. Their role underscores the importance of visualizations that engage and inform diverse audiences. (d) Front-line workers and at-risk groups, including patients, healthcare professionals, caregivers, essential workers, and the elderly, faced significant challenges during the pandemic. Visualizations for these groups provide critical insights into infection rates, safety protocols, resource allocation, and vaccination efforts, empowering informed decision-making and precautions. (e) The public, especially the most vulnerable, sought clarity amid the influx of daily guidelines. Visualizations served as a powerful tool, fostering trust in authorities and adherence to preventive measures. Transparent visualizations build trust and can help prevent future epidemics from escalating.

## DATA

### Health Data:

A main data source was related to the patients and the medical infrastructure. COVID-19 infection cases carry the most elementary information related to the pandemic reflecting the epidemiological triad: person, place, time; starting from the date of a positive test result of a patient, including the patient*’*s personal data, the type of the SARS-CoV-2 test, the list of direct contacts for each patient and the space-time context in which they met (e.g., travelers sharing transportation means).

The pandemic also required significant healthcare resources, overwhelming healthcare facilities and staff without suitable policies. Policymakers and health facility managers needed to monitor and adjust healthcare service capacity, considering key indicators, like the number of total/available ICU beds, general isolation capacity, and the availability of ventilators and ECMOs. More granular data included treatments given to patients and their locations across different wards and services. Many products were temporarily unavailable or only available to a limited extent during the pandemic. For example, at the onset of the pandemic, hospitals, surgeries, and care facilities suffered from supply bottlenecks for disinfectants, supply difficulties in procuring personal protective equipment for healthcare workers (masks, gloves, gowns, etc.) in quantity and in quality. Visualization approaches helped to monitor resources versus demands. From such patient and health infrastructure data, predictive models were also built to help policy-makers anticipate the effect of certain policies on the spread of the pandemic (e.g., making wearing a face mask mandatory).

### Derived data:

From raw data, additional data were then derived to meet various analytical needs. Aggregating infection cases by space and time helped understand disease prevalence and trends in specific regions. Contact information was modeled as a graph with space-time annotations, assisting infection prevention and control personnel in identifying patient clusters, superspreaders, and superspreading events. This enabled appropriate containment measures, such as stay-at-home orders for individuals or closedown orders for businesses.

### Socio-economic data:

Policymakers had to balance between the imperatives of public health and their societal impact. Nuanced decisions had to be made to preserve the economic fabric in the long run, while supporting the most fragile businesses in the short term. To this end, model builders also studied the economic impact of various policies, such as the maximum size of social gatherings.

## GOALS AND TASKS

The goals and tasks the data visualization community dealt with during the pandemic varied with the different stages of infection epidemiology present during COVID-19: low or high-level endemic, epidemic, pandemic, and seasonal patterns. From the domain perspective, we distinguish elementary and composite tasks. The categories below fit neatly into the general purposes of visualization,^7,8^ from exploratory and confirmatory analysis to presentation.

### Elementary Tasks:

E1) Understand socio-economic and political context; E2) Understand the R numbers (the average number of secondary infections generated by one infectious individual in either a fully or partially susceptible population) and population behavior; E3) Evaluate resource allocation and available capacity; E4) Communicate the findings and expose a line of reasoning for decisions.

### Composite Tasks:

C1) Contact tracing: find the frequency of the potential transmission routes and hot spots of the pathogen spreading and identifying other potential branches; C2) Understand pathogen spread and effect of interventions to control the transmission event (also disease-specific parameters); C3) Understand the correlation of SARS-CoV-2 exposure to the attack rate (or effective transmission), developing of symptoms (or COVID-19), hospitalization, need for ventilation and dead, respectively; note that asymptomatic individuals (who never develop symptoms) can also transmit the virus, which poses a unique challenge that should be acknowledged and addressed. C4) Evaluate specific mathematical or other models; C5) Evaluate the effectiveness of specific measures; C6) Quantify different risks, what-if scenarios; C7) Design and evaluate exit strategies.

## VISUAL MEDIA

Diverse visual media played a key role in conveying insights and empowering stakeholders across varied user communities, from interactive software systems to infographics.

*Interactive Software Systems,* often as visual dashboards, allowed users, from researchers to policymakers, to analyze live pandemic data, perform exploratory and confirmatory analyzes, and make collaborative decisions.

*Mobile Apps* provided essential information related to pandemics and user engagement, including contact tracking and real-time updates on case numbers and public health guidelines/preventive measures.

*Static Visualizations* effectively communicate key insights to decision makers and the public, offering concise overviews of complex data, without the need to interact and explore further.

*Interactive and Animated Visualizations,* including interactive time-lapse and video elements, effectively conveyed dynamic processes and trends, helping all communities understand and respond to the trajectory of the pandemic.

*Infographics and Data-Driven storytelling* through compelling visualizations, infographics, and multimedia presentations, data-driven storytelling captivated audiences, elucidating complex concepts and fostering empathy, making the data comprehensible.

## LESSONS LEARNED

The huge time pressure during the pandemic has often resulted in ad hoc visual analysis solutions. The data to visualize changed so quickly, we sometimes forgot to update color legends between dataset versions. It was also difficult to determine what features of the data were important. There was also not enough time to leverage visual scaffolding from visual encodings familiar to our clients toward more powerful and complex solutions. Although we are all proud to have helped advance our understanding of the pandemic, these projects are not necessarily our most polished products. Next time, we recommend to:

- *Start collaborations ahead of time,* to give the teams enough time to assimilate principles of team science, to build visual literacy, and to train and adapt continuously during the interpandemic period.
- *Document the data types and features* to visualize; a beginning is provided in this work.
- *Leverage visual scaffolding*^6^ by adopting visual encodings existent in the application domains.
- *Consider carefully the activities to support*^5^ with the right level of transparency and detail needed for different users.

There are also *good effects resulting from the pandemic*, such as: data visualization no longer being an afterthought, but a first-class citizen; increased literacy and acceptance of information density among our clients; and increased acceptance among domain experts of data visualization as a valid and exciting research field.

*In conclusion*, our exploration of data visualization needs during the COVID-19 pandemic illuminates the critical role visualizations played across various user communities and tasks. The pandemic prompted an unprecedented demand for insights derived from a deluge of heterogeneous data sources. Despite the challenges of time pressure, data stream dynamics, and communication barriers, our interdisciplinary teams navigated through projects spanning six countries, offering tailored solutions to diverse user groups.

From biomedical researchers to policymakers, journalists to frontline workers, and the general public, each community had distinct needs in understanding and responding to the pandemic. Visualization served as a powerful ally in conveying complex information, fostering transparency, trust, and informed decision-making. Our experiences underscore the importance of tailored visualizations in addressing the numerous challenges posed by global health crises.

As we reflect on our journey, we recognize the invaluable lessons learned and the importance to prepare for future emergencies. By documenting our experiences and insights, we aim to equip future practitioners with the knowledge and tools to navigate similar crises efficiently and effectively.

## Figures and Tables

**FIGURE 1.**
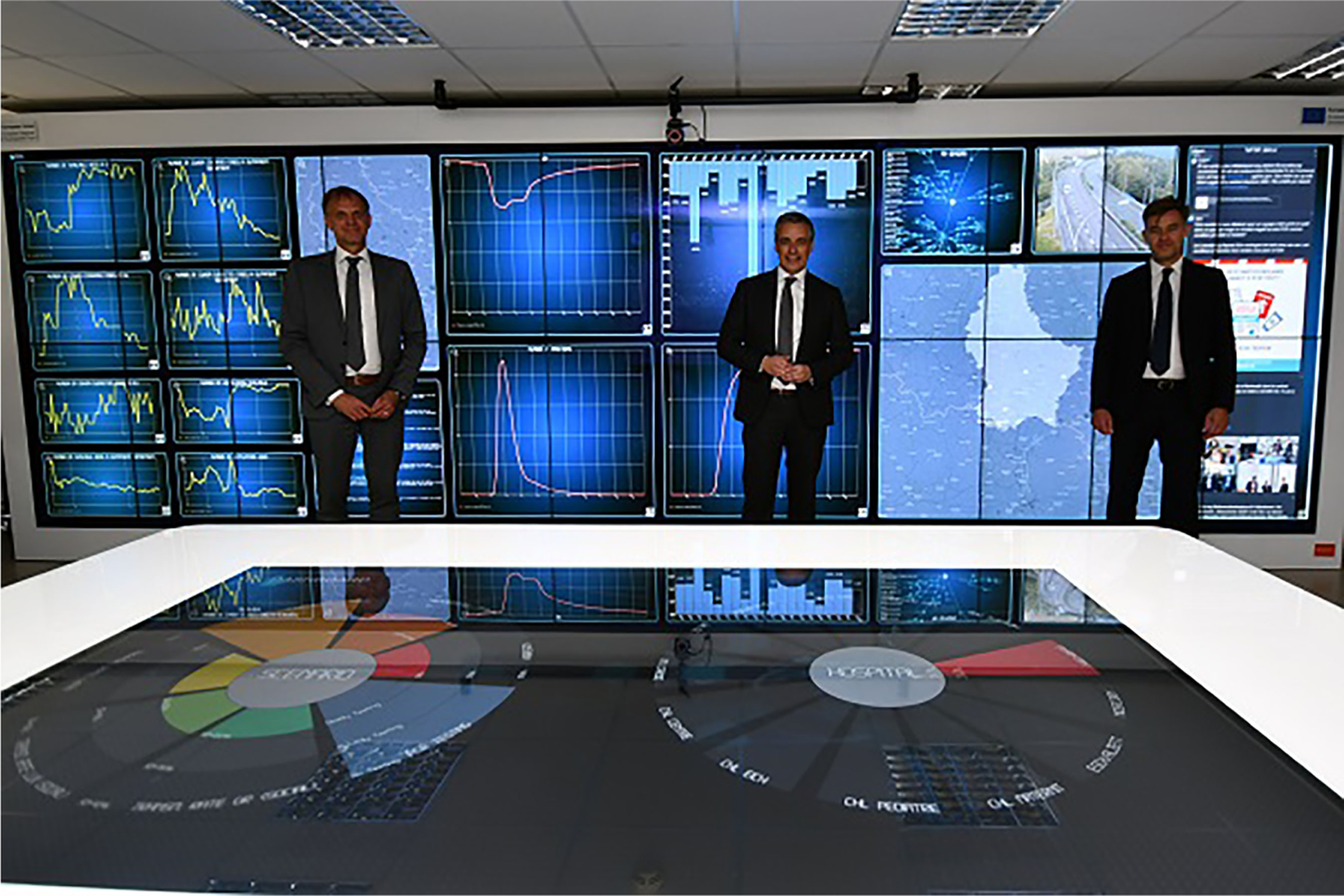
August 2020: Luxembourg ministers Franz Fayot and Claude Meisch view a pandemic Cross-Functional Dashboard demonstration, implemented in DEBORAH. A tabletop control panel sets the what-if scenario analyzed on the wall display.

**TABLE 1.** Media, clients, data, goals, and encodings across projects.

Project & Media	User Groups	Data	Goals/Tasks	Encodings
**COVis** Interactive web application	● Journalists ● Public	● COVID-19 statistics ● Public measures ● Predictive model data	● Identify national behavior & event impact ● Track temporal changes ● Interpret patterns	● Line, bar, and event charts ● Storytelling integration ● Logistic regression model
**CONTACT** Interactive web application	● Disease modelers ● Health experts ● Policymakers	● SEIR model outputs ● Transmission trees	● Analyze transmission patterns ● Evaluate quarantine impact ● Communicate contact tracing efficiency	● Transmission trees ● Network visuals ● Line graphs
**MOTIV** Interactive web application; screenshots for print/media	● Communication researchers ● Policymakers	● Health, demographic, and socio-economic data ● Social media ● Political context	● Understand public response ● Assess stay-at-home impact ● Leverage an inference model ● Share findings	● Choropleth and glyph maps ● Timelines, bar charts ● Custom encodings (AI inference model)
**B-FAST** Interactive web application	● Infection control staff ● Epidemiologists ● Healthcare professionals	● Patient data (vaccinations, stays, diagnoses)	● Detect outbreak indicators ● Identify transmission pathways	● Histograms ● Timelines ● Node-link diagrams ● Storyline visualizations
**HEADS** Interactive app	● Researchers ● Health officials ● Policymakers ● General public	● Aerosol emission data ● Virus dynamics ● Environmental parameters	● Assess infection risk ● Model aerosol spread ● Evaluate safety measures	● Line charts ● Animated aerosol simulations ● Traffic light risk system
